# Cancer Metabolomics and the Human Metabolome Database

**DOI:** 10.3390/metabo6010010

**Published:** 2016-03-02

**Authors:** David S. Wishart, Rupasri Mandal, Avalyn Stanislaus, Miguel Ramirez-Gaona

**Affiliations:** 1 Department of Computing Science, University of Alberta, Edmonton, AB T6G 2E8, Canada; rmandal@ualberta.ca (R.M.); avalyn@ualberta.ca (A.S.); miguel1@ualberta.ca (M.R.-G.); 2 Department of Biological Sciences, University of Alberta, Edmonton, AB T6G 2E8, Canada; 3 National Institute for Nanotechnology, 11421 Saskatchewan Drive, Edmonton, AB T6G 2M9, Canada

**Keywords:** cancer metabolomics, Human Metabolome Database (HMDB), metabolomics databases, oncometabolites, biomarkers

## Abstract

The application of metabolomics towards cancer research has led to a renewed appreciation of metabolism in cancer development and progression. It has also led to the discovery of metabolite cancer biomarkers and the identification of a number of novel cancer causing metabolites. The rapid growth of metabolomics in cancer research is also leading to challenges. In particular, with so many cancer-associate metabolites being identified, it is often difficult to keep track of which compounds are associated with which cancers. It is also challenging to track down information on the specific pathways that particular metabolites, drugs or drug metabolites may be affecting. Even more frustrating are the difficulties associated with identifying metabolites from NMR or MS spectra. Fortunately, a number of metabolomics databases are emerging that are designed to address these challenges. One such database is the Human Metabolome Database (HMDB). The HMDB is currently the world’s largest and most comprehensive, organism-specific metabolomics database. It contains more than 40,000 metabolite entries, thousands of metabolite concentrations, >700 metabolic and disease-associated pathways, as well as information on dozens of cancer biomarkers. This review is intended to provide a brief summary of the HMDB and to offer some guidance on how it can be used in metabolomic studies of cancer.

## 1. Introduction

Cancer is fundamentally a metabolic disease. As far back as 1927 [1], it was shown that cancer cells exhibit a distinct metabolic phenotype, consuming up to 200X more glucose than normal cells. This glycolytic phenotype, commonly known as the Warburg effect, has been found in nearly every type of cancer ever studied. However, interest in the influence of metabolism on cancer and its utility for cancer treatment largely waned in the 1960s and almost completely disappeared by the 1970s. The “re-discovery” of cancer as a metabolic disorder largely occurred in the last ten years [2,3]. This shift in thinking has mostly been due to the increased accessibility of metabolomics, the identification of useful cancer metabolite biomarkers and the discovery, via metabolomics, of “oncometabolites” [4]. Oncometabolites are endogenous metabolites that either initiate or sustain tumor growth and metastasis. The first oncometabolite to be identified was 2-hydroxyglutarate, a rare metabolite found in high concentrations in gliomas [3]. Since the discovery of 2-hydroxyglutarate many other oncometabolites have been identified including fumarate (renal cell carcinoma), succinate (paraganglioma), sarcosine (prostate cancer), asparagine (leukemia), choline (prostate, brain, breast cancer) and polyamines (most cancers). Almost all of these oncometabolites arise from, or are needed for, a number of key cancer-associated metabolic pathways, including aerobic glycolysis, glutaminolysis and one-carbon metabolism.

The discovery of oncometabolites (via metabolomics) has also been complemented with the discovery (also via metabolomics) of many other cancer-associated metabolites that could potentially serve as cancer biomarkers. These include numerous metabolite biomarkers found in serum [5], plasma [6], urine [7], saliva [8], and tissue samples [9]. Recently, volatile organic compounds (VOC) from breath and breath condensate have been found to be useful biomarkers of lung cancer [10,11]. Some examples of cancer-associated metabolite biomarkers are: phosphocholine, isoleucine, threonine, glutamate, histidine, acetoactetate, glycerol, mannose, phenylalanine and pyruvate in breast cancer [12,13,14]; taurine, lactate, choline, phenylalanine, isoglutamine, tyrosine, lipids, triglycerides, TCA cycle intermediates in colorectal cancer [15,16]; phosphatidylcholine, lysophosphatidylcholine, phosphocholine, glycerophosphocholine, and arachidonic acid in head and neck cancer [17].

Unfortunately, with so many cancer-associate metabolites being identified it is often difficult for scientists to keep track of which compounds are associated with which cancers. It is also challenging to track down information on the physiological role or the specific pathways that particular cancer-associated metabolites (or oncometabolites) may be affecting. Even more frustrating for metabolomics researchers and cancer researchers alike are the difficulties associated with identifying metabolites from NMR (nuclear magnetic resonance) or MS (mass spectrometry) spectra of metabolite mixtures [18]. What is clearly needed to address these challenges are comprehensive, open access databases that contain up-to-date referential information about metabolites, metabolic pathways, biomarkers and reference NMR, MS/MS (tandem mass spectrometry), and GC-MS (gas chromatography mass spectrometry) spectra for compound identification. Fortunately, a number of freely accessible metabolomics databases are starting to appear that offer at least some of these features. One such database is the Human Metabolome Database or HMDB [19,20]. The HMDB is currently the world’s largest and most comprehensive, organism-specific metabolomics database. It contains nearly 42,000 metabolite entries, more than 5000 normal and abnormal metabolite concentrations, nearly 800 metabolic and disease-associated pathways, and detailed information on dozens of cancer biomarkers. In this review, we will describe the HMDB in more detail and highlight some of its unique display features, search capabilities and data content attributes. We will also offer some guidance, selected case studies and simple examples on how to use the HMDB in metabolomic studies of cancer.

## 2. A Brief Overview of the HMDB

The Human Metabolome Database (HMDB) (www.hmdb.ca) contains comprehensive spectroscopic, quantitative, analytic and physiological information about human metabolites, their associated enzymes or transporters, their abundance and their disease-related properties. Since its first release in 2007 [19], the HMDB website has been accessed more than 10 million times and its associated papers cited nearly 3000 times. Broadly speaking, the HMDB contains three kinds of data: (1) chemical data; (2) clinical data; and (3) molecular biology/biochemistry data. The chemical data in the HMDB covers both water-soluble and lipid soluble metabolites as well as metabolites that would be regarded as either abundant (>1 uM) or relatively rare (<1 nM). The metabolite entries in HMDB are classified into three groups, “detected” metabolites [separated into two categories: (i) detected and quantified and (ii) detected but not quantified] and “expected” metabolites (those for which biochemical pathways are known or human intake/exposure is frequent but the compound has yet to be detected in the body).

**Figure 1:**
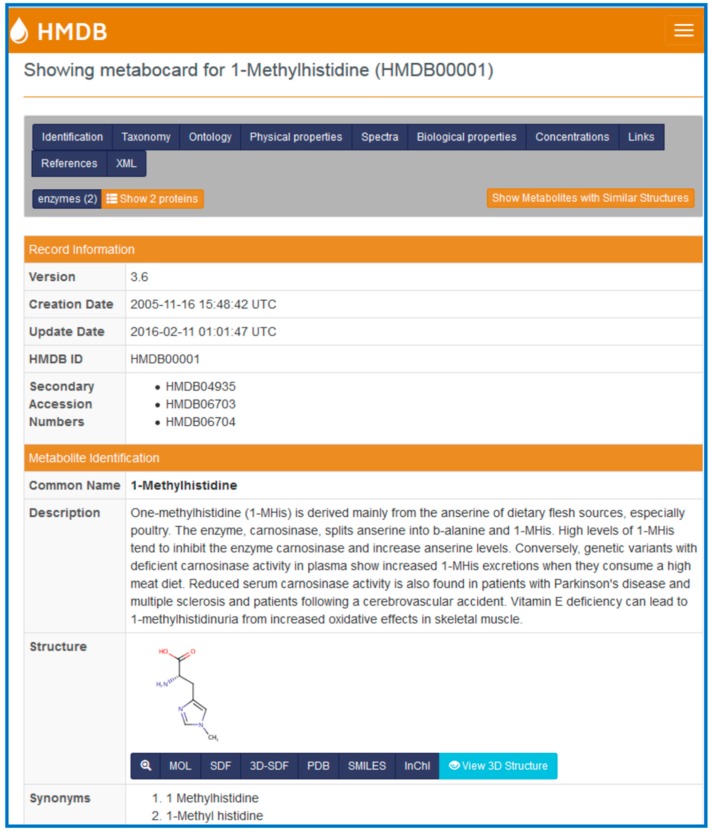
A screenshot of a MetaboCard (in the Human Metabolome Database (HMDB)) showing different categories or superfields.

Each metabolite in the HMDB is linked to a “MetaboCard”. Every MetaboCard contains 114 data fields with approximately two-thirds of the information being devoted to chemical or physico-chemical data and the other third devoted to biological or biomedical data. Each MetaboCard is divided into 14 distinctive categories or superfields with clearly demarcated titles (Figure 1). These superfields include: (i) record information; (ii) metabolite identification; (iii) chemical taxonomy; (iv) chemical ontology; (v) physical properties; (vi) spectra; (vii) biological properties; (viii) normal concentrations; (ix) abnormal concentrations; (x) associated disorders; (xi) external links; (xii) references; (xiii) enzymes; and (xiv) transporters. In addition to providing comprehensive numeric, sequence and textual data, each MetaboCard also contains hyperlinks to many other online databases (Kyoto Encyclopedia of Genes and Genomes (KEGG) [21], BioCyc [22], PubChem [23], Chemical Entities of Biological Interest (ChEBI) [24], PubMed, Protein Data Bank (PDB) [25], Universal Protein Resource (UniProt) [26], GenBank [23], Online Mendelian Inheritance in Man (OMIM) [23] and the Single Nucleotide Polymorphism Database (dbSNP)) [23]), abstracts, digital images and applets for viewing molecular structures and pathways. All the metabolites in HMDB are linked to more than 333,311 different synonyms. These metabolites are further connected to 721 non-redundant pathways, 6305 distinct enzymes, 110,000 SNPs as well as 617 metabolic diseases (genetic and acquired). Nearly 1400 compounds are also linked to experimentally acquired referential ^1^H and ^13^C NMR, MS/MS and GC-MS spectra. Concentration data (normal and abnormal values) for plasma, urine, CSF and/or other biofluids are also provided for a total of 5027 compounds. The entire database, including text, sequence, and structure and image data occupies nearly 20 GB of data—most of which can be freely downloaded. A detailed list of the HMDB content is shown in Table 1.

The HMDB is fully searchable with many built-in tools for viewing, sorting and extracting metabolites, biofluid concentrations, enzymes, genes, NMR or MS spectra and disease information. Detailed instructions on where to locate and how to use these browsing/search tools are provided on the HMDB homepage (www.hmdb.ca). As with most on-line databases, the HMDB supports standard text queries through a text-based search box located near the top right of each page. It also offers general database browsing using the drop-down menu of “Browse” button for metabolites, diseases, pathways, biofluids, metabolite classes, proteins and reactions located in the top left corner of HMDB menu bar. The Biofluids link in the Browse menu generates hyperlinked tables listing normal and abnormal concentrations of different metabolites for 23 different biofluids.

Located on the right of the Browse menu is the Search menu. Under Search, the ChemQuery (structure search) link allows users to draw (using a ChemSketch applet) or type in (using a SMILES string) a chemical compound and to search HMDB for chemicals similar or identical to the query compound. The Molecular Weight Query allows users to search for metabolites in the HMDB according to a molecular weight range. The Text Query supports a more sophisticated text search (partial word matches, case sensitive, misspellings, *etc.*) of the text portion of HMDB. HMDB’s Sequence Search allows users to conduct BLAST sequence searches of the over 5701 gene and protein sequences contained in HMDB. Both single and multiple sequence BLAST queries are supported. The Advanced Search link opens an easy-to-use relational query search tool that allows users to select or search over various combinations of subfields. The MS and MS/MS Searches allow users to submit Mass spectral files that will be searched against the HMDB’s library of MS and MS/MS spectra. This allows the identification of metabolites from mixtures via MS/MS spectroscopy. The 1D and 2D NMR Searches allow users to submit peak lists from ^1^H or ^13^C NMR spectra (both pure and mixtures) or 2D Total correlation spectroscopy (TOCSY) or ^13^C Heteronuclear single-quantum correlation spectroscopy (HSQC) spectra, respectively, and to have these spectra compared to the NMR libraries contained in the HMDB. This allows the identification of metabolites from mixtures via NMR spectroscopy.

Over the past three years, a significant effort has been undertaken to upgrade the number and quality of reference NMR, MS/MS and GC-MS spectra in the HMDB. In particular, hundreds of additional reference MS and NMR spectra were collected, assigned, and/or annotated by HMDB curators, whereas hundreds of additional annotated/assigned MS and NMR reference spectra were obtained from the BioMagResBank [27], METLIN [28] and MassBank [29]. As a result, the HMDB now contains approximately 3186 experimental ^1^H and ^13^C NMR spectra for 1381 compounds. It also contains 1200 MS/MS (Triple-Quad) spectra at three different collision energies for 1250 pure compounds. Additionally, GC-MS reference data/spectra are now available for 1220 compounds. The HMDB’s spectral search utilities allow both pure compounds and mixtures of compounds to be identified from their MS or NMR spectra via peak matching algorithms that were developed in-house. All NMR spectral assignments are now available for download in an NMR-STAR format [27]. This format captures all relevant spectral features, spectral collection conditions, assignments and chemical structure information. NMR spectra are also available as raw (FID) files and as simple images (PNG format). In addition to NMR spectra, nearly all MS/MS spectra in the HMDB are now available in mzML format. The mzML format is rapidly becoming the preferred format for MS data exchange as it robustly captures all relevant spectral features, MS spectral collection conditions and associated annotations. All MS spectra in the HMDB are also available as simple images (PNG format) and as simple mass list files.

Perhaps the most relevant features of the HMDB from the perspective of a medical researcher are its rich content and extensive linkage to metabolic diseases, to normal and abnormal metabolite concentration ranges (in many different biofluids) and pathways associated with many diseases of interest. Over the past four years, several comprehensive studies conducted by us have led to the experimental validation and accurate concentration measurements of thousands of compounds in normal cerebrospinal fluid [30], serum [31], urine [32] and saliva [33]. Four additional databases, DrugBank [34], T3DB [35], SMPDB [36] and FooDB (www.foodb.org) are also part of the HMDB suite of databases. DrugBank contains equivalent information on 8070 drug and drug metabolites, T3DB contains information on 3673 common toxins and environmental pollutants, SMPDB contains pathway diagrams for more than 1700 metabolic and disease pathways, while FooDB contains equivalent information on 26,619 food constituents and food additives.

## 3. Cancer Metabolism Pathways in the HMDB

Currently, the HMDB contains links to nearly 80 KEGG [21] pathways and 721 SMPDB [36] pathways. Collectively, these two sets of pathway collections are linked to 1124 different metabolites in the HMDB. While the KEGG pathways are perhaps better known, the SMPDB pathways in the HMDB contain considerably more information and cover a far wider range of biological actions. In particular, the SMPDB pathways in the HMDB include metabolic, metabolite signaling, disease process, drug action and drug metabolism pathways. All of the SMPDB pathway diagrams are richly colored, highly detailed, zoomable, comprehensively annotated and fully-hyperlinked. Each SMPDB pathway explicitly shows all relevant compound structures, all protein quaternary structures, enzyme cofactors and subcellular compartments where specific reactions are known to take place. All chemical structures in these pathway maps are hyperlinked to HMDB MetaboCards and all enzymes are hyperlinked to UniProt data cards. Each pathway diagram in the HMDB is fully searchable (via PathSearch) and each pathway has a short synoptic description. All of the pathway maps in the HMDB (and SMPDB) have been created using an online drawing tool called PathWhiz [37]. As a web server, PathWhiz is accessible from almost any place and compatible with essentially any operating system. It also houses a public library of more than 1700 pathways and thousands of pathway components that can be easily viewed and expanded upon by its users. PathWhiz allows users to readily generate biologically complex pathways by using a specially designed drawing palette to quickly render metabolites (including automated chemical structure generation), proteins (including quaternary structures, covalent modifications and cofactors), nucleic acids, membranes, subcellular structures, cells, tissues and organs. Because PathWhiz pathways can be saved in BioPAX, SBGN-ML and SBML formats, as well as PNG, PWML, HTML image map or SVG files, it means that all of the HMDB (and SMPDB) pathways generated via PathWhiz are easy to view, easy to share and are compatible with almost any third-party pathway software analysis or pathway viewing tool. Currently, the HMDB has 226 (PathWhiz-generated) disease-associated pathways, including nearly 30 that are cancer-associated. These include metabolic pathways widely implicated in cancer such as the Warburg effect (aerobic glycolysis), glutaminolysis and one-carbon metabolism. They also include pathways depicting the action of four recently discovered oncometabolites including 2-hydroxyglutarate, fumarate, succinate and sarcosine. In addition to these cancer metabolic pathways, the HMDB also has seven cancer drug metabolism pathways (for doxorubicin, ifosfamide, tamoxifen, *etc.*) and 12 cancer drug action pathways (methotrexate, cyclophosphamide, bevacizumab, cetuximab, irinotecan, ifosfamide, *etc.*). Below, we will describe in detail some of the more pertinent or interesting cancer pathways contained in the HMDB.

### 3.1. The Warburg Effect

**Figure 2:**
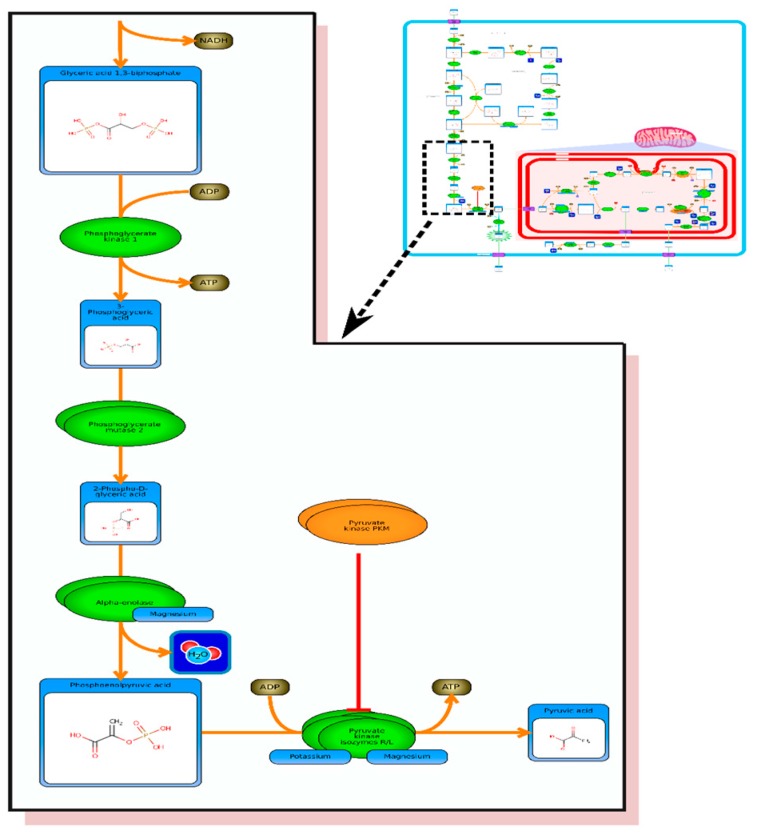
Inset: A Picture of HMDB’s Warburg effect pathway (generated via PathWhiz). Expanded region shows a portion of the pathway in greater detail.

In the HMDB, the Warburg effect pathway is linked to (and accessible via) a large number of common metabolites, including glucose, glucose-6-phosphte, fructose-6-phosphate, pyruvate, lactic acid, and many other well-known molecules. A picture of HMDB’s Warburg effect pathway (generated via PathWhiz) is shown in Figure 2. The Warburg effect [1] refers to the phenomenon that occurs in most cancer cells where instead of using glucose to generate energy (ATP) via the Krebs cycle in the mitochondria, cancer cells direct glucose to undergo lactic acid fermentation in the cytosol. However, in cancer cells, glucose is not used for energy production but instead it is used for lactic acid production and cell constituent production (*i.e.*, amino acids, nucleic acids, lipids) that are needed for rapid cell division. In normal cells lactate production is reserved for anaerobic conditions. What is unusual about this glycolytic pathway is that is largely aerobic, occurring even when oxygen is plentiful. Hence, the Warburg effect is often called “aerobic glycolysis”. The Warburg Effect is thought to be the result of multiple mutations to oncogenes and tumor suppressor genes, all of which lead to this common glycolytic phenotype [2]. Because aerobic glycolysis is so energetically inefficient, cancer cells must consume huge amounts of glucose—up to 200X more than normal cells. Cancer’s “sweet tooth” is the basis to glucose imaging via positron emission tomography (PET) [2]. The Warburg effect is thought to be an ancient cellular adaptation to surviving and propagating in low-oxygen environments, which may have been found in freely moving organisms in ancient oceans [2]. Low oxygen environments are also commonly found within tumors. It is also believed that the Warburg effect is the result of cancer genes shutting down the mitochondria (leading to cytosolic metabolism), or a mechanism to aid in cell survival and proliferation via increased production of the molecular constituents of the cell [2]. The Warburg effect is linked to numerous other pathways, including growth factor stimulation, mTOR signaling and transcriptional activation.

### 3.2. The Oncogenic Action of 2-hydroxyglutarate

**Figure 3:**
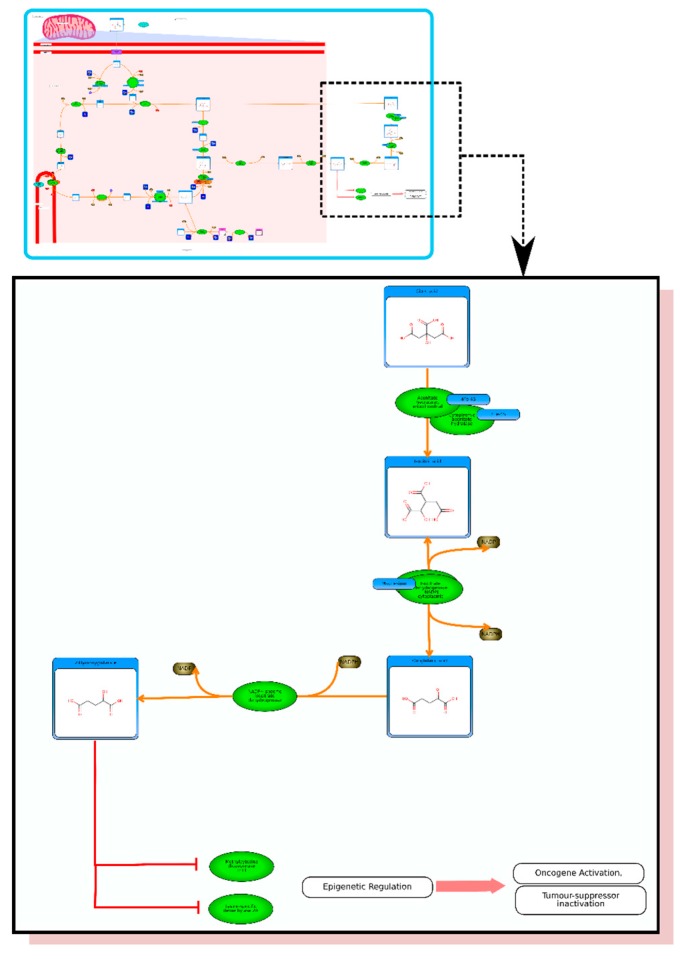
Inset: A picture of “The oncogenic action of 2-hydroxyglutarate” pathway in HMDB (generated via PathWhiz). Expanded region shows a portion of the pathway in greater detail.

If one enters the term “2-hydroxybutyrate” in HMDB’s text search, the MetaboCard for this compound will instantly appear. Scrolling down the web page to this metabolite’s pathway section will reveal a link to a pathway entitled “The oncogenic action of 2-hydroxyglutarate” (Figure 3). 2-hydroxyglutarate was the first oncometabolite (or cancer-causing metabolite) to be formally named or identified [3]. While there are many known chemical carcinogens (exogenous or xenobiotic chemicals), there are relatively few endogenous metabolites that appear to be directly associated with an increased risk of cancer. 2-hydroxyglutarate is the product of gain-of-function mutations in the cytosolic and mitochondrial isoforms of isocitrate dehydrogenase (IDH). IDH is part of TCA cycle and is generated in high abundance when IDH is mutated. More recently, it has been found that overexpression of phosphoglycerate dehydrogenase (PHGDH), which is genomically amplified in tumors including breast cancer and melanoma, also produces excess quantities of 2-hydroxyglutarate [38]. 2-hydroxyglutarate is sufficiently similar in structure to 2-oxogluratate (2OG) that it is able to inhibit a range of 2OG-dependent dioxygenases, including histone lysine demethylases (KDMs) and members of the ten-eleven translocation (TET) family of 5-methylcytosine (5mC) hydroxylases [39]. In turn, this leads to modulations of the hypoxia induced factor (HIF)-mediated hypoxic response and alterations in gene expression through global epigenetic remodeling. The net effect is that 2-hydroxyglutarate causes a cascading effect that leads genetic perturbations and malignant transformation. Individuals with inborn errors in the IDH sequence are known to have very high rates of cancer. Likewise, people with glioma (a type of brain cancer) have been found to have elevated IDH levels as well [3,39]. However, targeting the IDH enzyme is proving to be difficult.

### 3.3. The Oncogenic Action of Fumarate & Succinate

**Figure 4:**
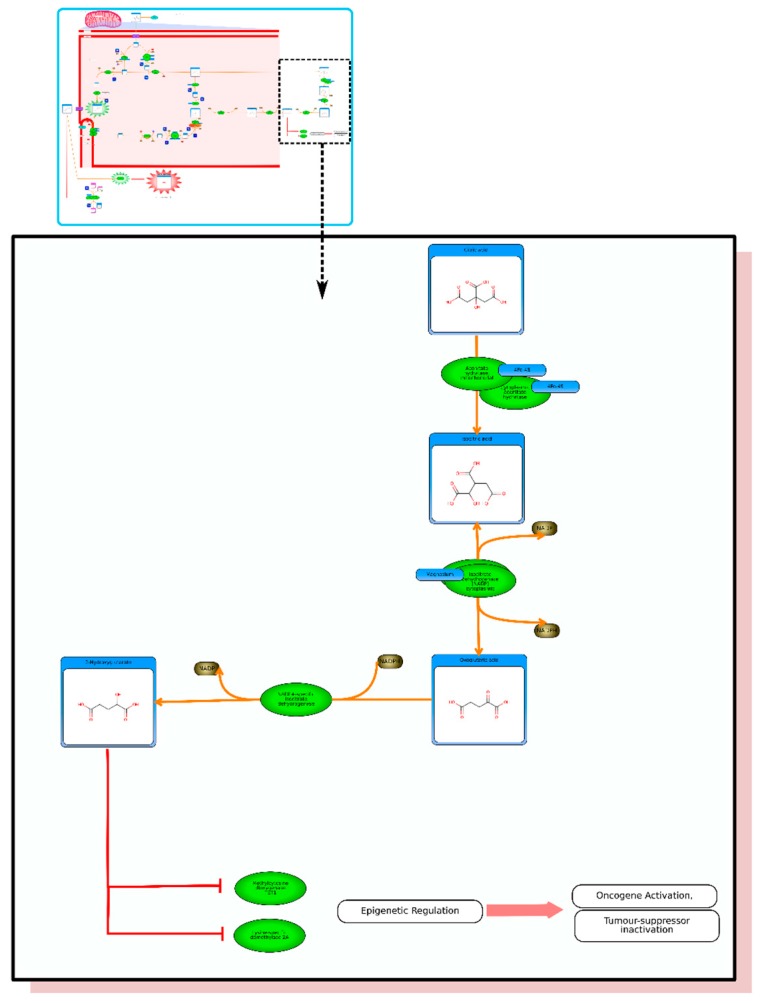
Inset: A picture of “The oncogenic action of fumarate” pathway in HMDB (generated via PathWhiz). Expanded region shows a portion of the pathway in greater detail.

**Figure 5:**
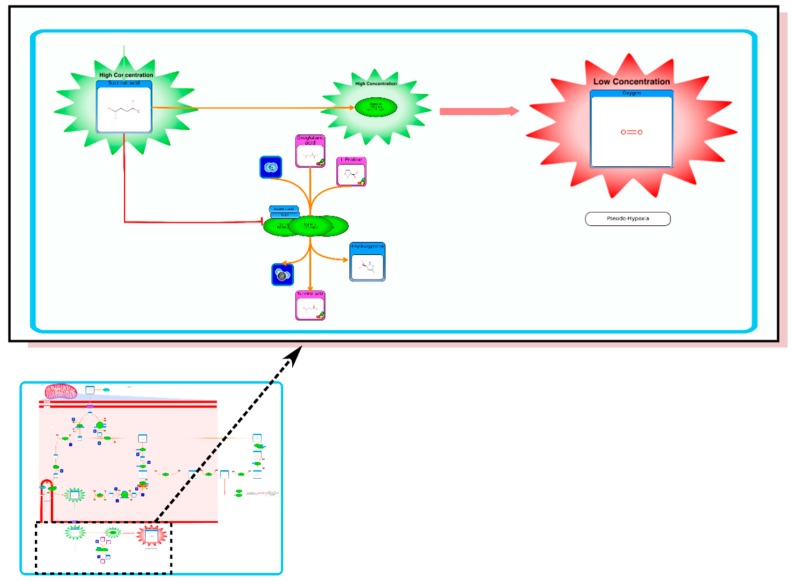
Inset: A picture of “The oncogenic action of succinate” pathway in HMDB (generated via PathWhiz). Expanded region shows a portion of the pathway in greater detail.

Entering “fumarate” or “succinate” in HMDB’s text search will allow one to scroll to the pathways depicting the oncogenic action of fumarate and succinate (Figure 4 and Figure 5). Both of these molecules are now considered oncometabolites and both have been shown to sustain the growth of tumors. The pathways depicted in HMDB show how these compounds interact with a number of important proteins that regulate transcription, oxygen tension, mitochondrial function and aerobic/anaerobic metabolism. In many tumors, oxygen availability becomes limited (hypoxia) very quickly due to rapid cell proliferation and limited blood vessel growth. The major regulator of the response to hypoxia is the HIF transcription factor (HIF-alpha). Under normal oxygen levels, protein levels of HIF-alpha are very low due to constant degradation, mediated by a series of post-translational modification events catalyzed by the prolyl hydroxylase domain-containing enzymes PHD1, 2 and 3, (also known as EglN2, 1 and 3) that hydroxylate HIF-alpha and lead to its degradation [39]. All three of the PHD enzymes are inhibited by both succinate and fumarate. Under hypoxic conditions, HIF-alpha escapes hydroxylation and HIF-alpha becomes activated, which leads to aerobic glycolysis and tumorigenesis. The association between fumarate/succinate and cancer was noticed by the high rates of fumarate hydratase and succinate dehydrogenase mutations in certain cancers [39]. Fumarate hydratase (FH) is a housekeeping gene, but mutations in this gene allow for fumarate to accumulate and cross the mitochondrial barrier and into the cytosol through a transport protein called the dicarboxylate carrier. The same substrate accumulation can happen with mutations to succinate dehydrogenase, leading to high cytosolic levels of succinate. Once in the cytosol, fumarate and/or succinate are able to inhibit the activity of the PHD1, 2 and 3 enzymes, thereby leading to HIF-alpha activation and, eventually, cancer.

## 4. Cancer Biomarkers in the HMDB

We believe that one of the most important and unique features of the HMDB is the information it contains on metabolite concentrations (both normal and abnormal) along with their disease associations and tissue locations. Over the past 10 years, the HMDB’s literature curation team has added an extensive body of data that links a wide range of diseases to normal and abnormal metabolite concentration ranges for more than 30 different biofluids and tissue types. This compilation of reference ranges or reference concentrations, abnormal concentrations and disease associations has made the HMDB particularly useful for clinical chemists and physicians.

Currently the HMDB lists a total of 617 different diseases along with their corresponding abnormal metabolite concentrations (typically quoted in μM or μM/mM creatinine). Any given disease may have as little as one or as many as 30 abnormal metabolites listed in the database. Out of these 617 diseases, 37 are for different types of cancers, including breast, kidney, colon, endometrial, bronchial, pancreatic, prostate, ovarian, bladder, esophageal, lung, stomach, cervical, oral, colorectal, thyroid, cholangiocarcinoma and hepatocellular carcinoma. A total of 280 unique metabolites (biomarkers) are associated with these cancers and they are present in 10 different kinds of biofluids (blood, urine, CSF) and tissues. All these cancer biomarkers are searchable via the HMDB using the “Browse” function and looking under the “Diseases” submenu located on the left side of the HMDB menu bar. This information can also be found using the keyword search by typing the words “cancer and disease” in the text search box that is available from the drop-down Search menu. For each type of cancer, the corresponding biomarkers/metabolites, abnormal concentration values, age, sex and associated PubMed or other literature references are provided.

It is important to note that the collection of cancer-associated metabolite markers in the HMDB is still far from complete. Given that new papers reporting dozens of new cancer-associated metabolite biomarkers are appearing almost every day, it is now impossible for the HMDB curation team to stay current. However, over the next two years, the HMDB will be providing much more detailed information regarding biomarker sensitivity and specificity values as well as “area under the receiver operating characteristic” (AUROC) curve data for many of the most robust or clinically validated metabolite biomarkers.

## 5. Using the HMDB in Cancer-Related Metabolomics Studies

One of the biggest challenges in metabolomics involves the identification and/or quantification of compounds. In most untargeted metabolomic studies, compounds must be identified by comparing the accurate molecular weight (*m*/*z*), retention time, NMR, GC-MS and/or LC-MS/MS spectrum of the “unknown” or “query” compound with some kind of database containing the molecular weights or spectra of known metabolites [18]. Finding a good or, ideally, a perfect match between the query and the database provides the necessary (but not sufficient) evidence that the compound has been properly identified.

While many cancer metabolomics researchers typically want to search the largest possible database (*i.e.*, PubChem for *m*/*z* data searches, the NIST database for GC-MS data searches or METLIN for LC-MS/MS data searches), for their metabolomic queries, this is often not the best choice. This is because these databases do not limit their compound collections or spectral data sets to human-only or mammalian-only compounds. For instance, PubChem contains 61 million compounds, but less than 0.5% of its chemicals are metabolites, natural products or compounds that could possibly be found in humans and/or other mammals. The other 99.5% have never left the laboratory or were never produced in sufficient quantity to be detected in any biological sample. In the case of the NIST GC-MS database, only a small fraction (<5%) of the compounds in their vast GC-MS library appear to be metabolites or natural products that could possibly be found in humans or other mammals. A similar issue has emerged for the METLIN database, as no information about the possible biological origin of its compounds or their associated MS/MS spectra is provided. As a result, it is not uncommon for researchers doing untargeted metabolomic studies on mice, for instance, to report “impossible” compound identifications, such as exotic spices, rare marine compounds or banned performance enhancing drugs—simply because they naively chose to only look at the largest possible chemical or spectral database to do their compound identification. In other words, for metabolomics to work, the database needs to match the organism.

In this regard, the HMDB is actually an ideal database for cancer metabolomic studies. This is because the compounds and the reference spectra contained in the HMDB are limited to compounds that are known, or widely expected, to be in humans. These include more than 29,000 endogenous metabolites, thousands of common food constituents, hundreds of microbial metabolites and dozens of high-abundance environmental chemicals or contaminants. In many cases, these same compounds are also found in mice, rats or other mammalian models used for cancer research. The HMDB is also unique with regard to the diversity of its spectral reference data. Unlike the BioMagResBank (which is limited to NMR-only data) or METLIN (which is limited to LC-MS data), the HMDB houses extensive NMR, GC-MS and MS/MS spectral data. Furthermore, the HMDB provides well-supported *m*/*z* and spectral search functions that allow users to easily submit, view and assess their spectral matches. Users can also select between expected (33,000), detected (7000) or all (42,000) metabolites in the HMDB for their MS search routines. A simple adduct calculator generates more than 25 possible adducts (containing Na+, K+, NH_4_+ and dimer adducts) for three different modes (positive ion, negative ion, neutral) for each compound, creating an effective database of more than one million masses for high resolution parent ion matching. The HMDB’s NMR search utilities allow users to submit peak lists from 1D ^1^H or ^13^C NMR spectra (both pure and mixtures) or 2D TOCSY or 2D ^13^C HSQC spectra, respectively, and to have these spectra compared to the NMR libraries contained in the HMDB. This allows facile identification of metabolites from mixtures via NMR spectroscopy. In particular, the HMDB’s NMR search utilities were used for one of the first metabolomic studies on cancer cachexia using urine samples [40]. These unique search functions and large spectral libraries permitted the identification of a number of predictive biomarkers for cancer-induced cachexia that could not be identified via commercial software or any other source. In addition to their utility in cancer biomarker applications, the NMR spectral libraries in the HMDB have recently been used to develop an automated tool for NMR spectral deconvolution, called Bayesil [41]. Bayesil is able to take raw NMR spectra from 500 and 600 MHz NMR instruments and automatically phase, reference, baseline correct and identify as well as quantify up to 60 metabolites from complex mixtures such as serum, cerebrospinal fluid and saliva. This new tool is now being used by a number of laboratories to look for pre-cancerous biomarkers for colon cancer.

While the HMDB’s spectral libraries are very helpful for compound identification, it is the HMDB’s associated chemical and biochemical information that is perhaps far more useful. In other words, the HMDB allows users to not only identify or confirm the existence of a compound, it also allows users to almost understand its biological or biochemical context. In this regard, the HMDB appears to be unique among all existing metabolomic or reference spectral databases. In particular, each compound in the HMDB has information regarding its name(s), synonyms, 2D and 3D structure, molecular weight, general description, references, biological activities, probable origin, structural properties, chemical classification and chemical similarity. Many compounds in the HMDB also have additional information regarding their known chemical reactions, associated enzymes or transporters, biological pathways, tissue or biofluid locations, disease associations and normal/abnormal concentrations. This rich information content is also quite easily mined and even more easily integrated into more advanced analytical tools for metabolomics. Indeed, the HMDB is now the primary reference source for many of the functions offered through MetaboAnalyst [42]. MetaboAnalyst is a web-based package for performing comprehensive metabolomic analysis and biomarker identification that is now accessed by more than 75,000 users a month. Much of the success of MetaboAnalyst appears to be tied to its tight linkage to the rich information in HMDB.

## 6. A Sample Workflow Using HMDB and SMPDB

**Figure 6:**
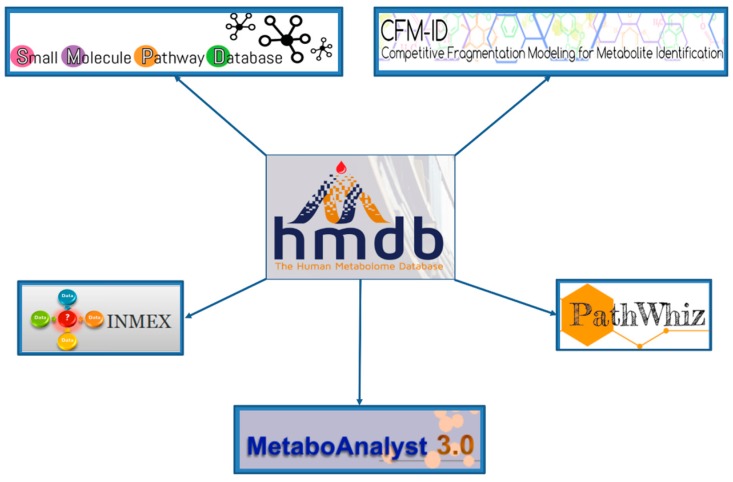
The “hub-and-spoke” linkage of HMDB with several metabolomics analysis tools.

The HMDB serves as a hub to many online metabolomics and metabolism resources. As noted already, it is linked to several metabolomic analysis tools such as MetaboAnalyst [42], PathWhiz [37], SMPDB [36], CFM-ID [43,44] and INMEX [45]. These tools use the HMDB’s data resources to facilitate metabolomic data interpretation, analysis and visualization. This “hub-and-spoke” linkage is shown in Figure 6. To better illustrate how these linkages occur we will demonstrate how the HMDB and SMPDB could be used in a cancer metabolomics study. For this example, let us assume that a high resolution MS experiment has been completed on CSF (cerebrospinal fluid) collected from a set of 30 patients with glioma and another set of 30 patients with no cancer symptoms (but with suspected meningitis, spinal fractures, *etc.*). In the positive ion mode, peaks with the following *m*/*z* values were found to be significantly increased in the cancer patients: 149.0444, 117.0183, 119.0339, 91.0388 while the peaks with the following *m*/*z* values were found to be significantly decreased in the cancer patients relative to the non-cancer “controls”: 147.0763, 193.0341, 308.0911.

**Figure 7:**
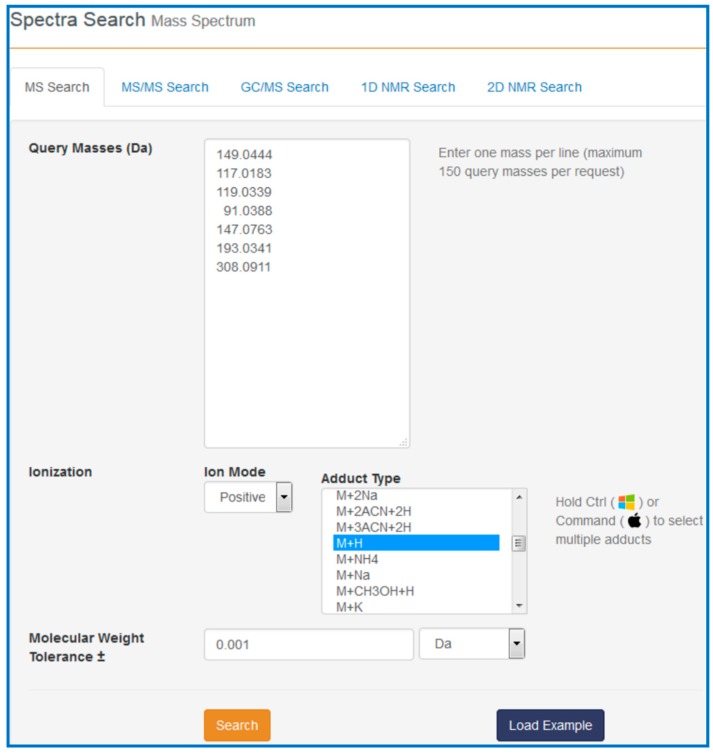
A sample screen shot for MS-Search query in HMDB.

Our task is to find out what these compounds are, how they might be involved in cancer and with which pathways they are associated. To answer these questions, go to the HMDB website (www.hmdb.ca) and click on the “Search” tab (at the top of the HMDB menu bar). The drop down menu will display several search options including sequence, text, spectral and molecular weight searches. For this example select the “MS Search” option. Once this is selected, type in the *m*/*z* values (listed above) into the “Query Masses” box. Make sure the Ion Mode is marked positive and the molecular weight tolerance is set to 0.001 Da. Choose the adduct type to be “M + H”. See Figure 7 for a sample screen shot of what the query should look like. Press the search button. In a few seconds a list of top scoring hits in the HMDB will be displayed. In some cases five to six metabolites with identical masses (isobaric compounds) will be displayed.

From the list of top matches, click on any HMDB hyperlink under the “Compound” column. For instance, if you click HMDB00606 this will take you immediately to the HMDB metabocard for d-2-hydroxyglutarate. Reading through the description you will see that d-2-hydroxyglutarate has been implicated as an oncometabolite as well as a key metabolite in an inborn of metabolism (d-2-hydroxyglutaricaciduria). Scrolling down this information card, you will see its structure, its synonyms, its chemical classifications, its concentrations in different biofluids, a list of pathways that it is involved in and the enzymes or transporters that bind it or act on it. For this example we will limit our discussion to molecules that have moderately high concentrations (>1 uM) in known biofluids and which have known links to cancer metabolism. This information can be gleaned by clicking on each of the links from our MS search and reading the material in each HMDB metabocard. To save time, the final list of seven metabolites is: d-2-hydroxyglutarate (increased), fumarate (increased), succinate (increased), lactate (increased), glutamine (decreased), isocitrate (decreased) and glutathione (decreased).

Identifying compounds using only *m*/*z* data is always a bit risky. If we had MS/MS data for these compounds we could have searched for similar MS/MS spectra via HMDB’s “MS/MS Search” option or we could have gone to the CFM-ID website and performed a similar kind of MS/MS spectral search. Finding matching MS/MS spectra (either observed or predicted) to our query MS/MS spectra would have given us additional supporting evidence regarding the actual identity of these compounds.

**Figure 8:**
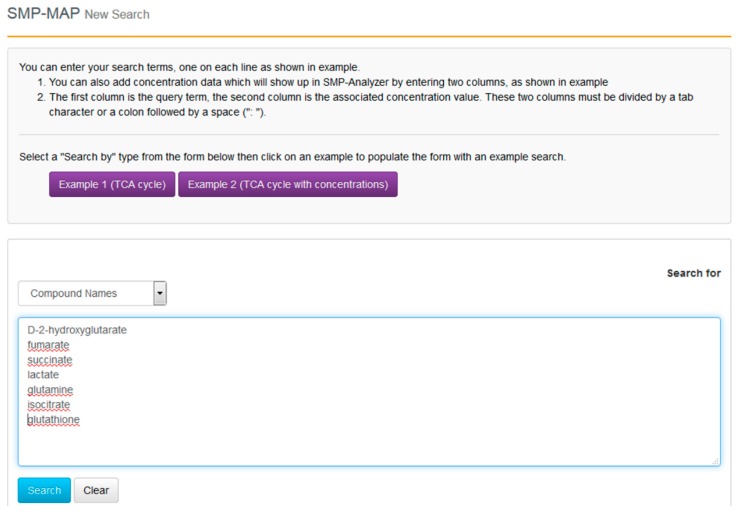
A sample screen shot for SMP-MAP Advanced Search query in SMPDB.

With the compounds identified and their clinical context more clearly delineated, we can now proceed to go to SMPDB and get a better picture of their molecular biological or biochemical context. SMPDB is a pathway database that uses HMDB as a reference “hub”. It also supports some useful pathway visualization tools. For this example go to the SMPDB website (www.smpdb.ca) and click on the “Search” tab (at the top of the SMPDB menu bar). The drop down menu will display several search options including sequence, text and molecular weight searches. For this example select the “SMP-MAP Advanced Search” option. Once this is selected, type in the list of compounds names (d-2-hydroxyglutarate, fumarate, succinate, lactate, glutamine, isocitrate, glutathione) in a single column. See Figure 8 for a sample screen shot of what the query should look like. Press the search button. In a few seconds a list of disease pathways associated with these metabolites should appear. On the top menu bar you should see a list of seven pathway categories (All, Metabolic, Physiological, *etc.*). On the far right tab “Disease Pathways” should be visible. If you click on that hyperlink a list of 175 Disease pathways should be visible. One of the top hits will be a pathway entitled “The oncogenic action of 2-hydroxyglutarate”. Clicking on any of these pathway images and zooming into each pathway using the JavaScript widgets should allow you to explore the pathway in more detail and gain an understanding of how 2-hydroxyglutarate is produced, its likely mechanism of action and the various upstream and downstream products of its metabolism and transport.

## 7. Conclusions and Future Directions

Thanks to metabolomics, the links between cancer and metabolism are becoming increasingly clear. These connections are opening the door to the discovery of new cancer mechanisms, unexpected cancer targets and novel cancer therapies. The increased interest in metabolomics by cancer researchers is also leading to a flood of novel, metabolite-based cancer biomarkers that could be used in the prediction, monitoring and diagnosis of cancer. The HMDB is attempting to capture as much of this information as possible and its curation team is working hard to make these data current and freely available to all through pathways, detailed compound descriptions, metabolite concentrations and a rich collection of references. In addition to its role as a biochemical information hub, the HMDB is also serving a key role in supporting metabolite annotation, identification and analysis for a large number of metabolomics researchers. The fact that it is a human or mammalian-specific database makes the HMDB particularly useful for cancer-based metabolomics. This is because it prevents possible mis-identifications or the detection of false positives from “contaminating” compounds that could not possibly have a biological or even environmental origin. The rich spectral information in the HMDB along with its complementary chemical, biological and biochemical information has led to its use in a number of cancer biomarker studies as well as its use in a number of other programs (Bayesil and MetaboAnalyst) that are increasingly being used in cancer research. As the field of metabolomics evolves, the HMDB will evolve with it. With increasingly more metabolomic studies being done on tissues [46,47], hair [48], volatiles in breath [49] and other less commonly studied biofluids (such as sweat and tears) future updates to the HMDB will, no doubt, include far more data on these important biological matrices. Likewise, as more and more data is being published on metabolic flux analysis [50] and isotope enrichment for metabolic dynamics [51], especially in cancer research, the HMDB (and allied resources) will also begin to capture and display these data. Certainly as the HMDB continues to expand and improve, especially with regard to its tracking of biomarkers, its planned inclusion of flux analysis data, and its growing number of cancer-associated pathways, its utility in cancer research and cancer-based metabolomics will continue to increase.

## Figures and Tables

**Table 1:** Content of the Human Metabolome Database (HMDB) 3.6.

Database Feature	HMDB (Version 3.6)
Number of metabolites	41,993
Number of unique metabolite synonyms	333,311
Number of compounds with disease links	3105
Number of compounds with biofluid or tissue concentration data	5027
Number of compounds with chemical synthesis references	1943
Number of compounds with experimental reference 1H and or 13C NMR spectra	1381
Number of compounds with reference MS/MS spectra	1250
Number of compounds with reference GC-MS reference data	1220
Number of human-specific pathway maps	721
Number of compounds in Human Metabolome Library	750
Number of HMDB data fields	114
Number of predicted molecular properties	10
Metabolite search/browse	yes
Pathway search/browse	yes
Disease search/browse	yes
Chemical class search/browse	yes
Biofluid browse	yes
Metabolite library browse	yes
Protein/transporter browse	yes
